# The efficacy of magnesium sulfate loading on microalbuminuria following SIRS: One step forward in dosing

**DOI:** 10.1186/2008-2231-20-74

**Published:** 2012-10-31

**Authors:** Bahador Mirrahimi, Hadi Hamishehkar, Arezo Ahmadi, Mohamad Reza Mirjalili, Mostafa Aghamohamadi, Atabak Najafi, Mohammad Abdollahi, Mojtaba Mojtahedzahed

**Affiliations:** 1 Department of Clinical Pharmacy, Faculty of Pharmacy, Tehran University of Medical Sciences, Tehran, 1417614411, Iran; 2 Department of Clinical Pharmacy, Faculty of Pharmacy, Tabriz University of Medical Science, Tabriz, 1476651664, Iran; 3 Department of anesthesia and intensive care, Sina hospital, Tehran University of Medical Science, Tehran, 1136746911, Iran; 4 Department of anesthesia and intensive care, Faculty of Medicine, Shahid Sadoghi University of Medical Science, Yazd, 8915173143, Iran; 5 Sina hospital, Tehran University of Medical Science, Tehran, 1136746911, Iran; 6 Department of Toxicology and Pharmacology, Faculty of Pharmacy, and Pharmaceutical Sciences Research Center, Tehran University of Medical Sciences, Tehran, 1417614411, Iran

**Keywords:** Magnesium, Microalbumin, TNF-α, Oxidative stress, Trauma, Critical care

## Abstract

**Backgrounds:**

Magnesium has been known for its antioxidative and antiinflammatory properties in many studies. In this study two dosing regimens of magnesium were compared with a placebo control group in order to investigate safety and efficacy of high doses of intravenous magnesium sulfate infusion on critically ill trauma patients. Inflammatory and oxidative factors were measured in this trial.

**Methods:**

45 trauma patients with systemic inflammatory response syndromes (SIRS) were randomly assigned into 2 treatment and one placebo groups. The high dose group received 15 g MgSO_4_, low dose group received 7.5 g of MgSO_4_ over 4 hour infusion, and placebo group received saline alone. The initial and post magnesium sulfate injections levels of tumor necrosis factor alpha (TNF-α), total antioxidant power and lipid peroxidation were measured after 6, 18 and 36 hours. The pre-infusion along with 6 and 36 hour level of microalbuminuria were also determined.

**Results:**

Repeated measurements illustrated that there was no significant difference in TNF-α, total antioxidant power and lipid peroxidation levels among groups during the period of analysis. The microalbuminuria at 36 hour post infusion of high dose group was lower than that of control group (p = 0.024). Patient’s mortality (28 day) was similar among all treatment groups. Both magnesium infusion groups tolerated the drug without experiencing any complications.

**Conclusion:**

No evidence for antioxidative and antiinflammatory effects of magnesium in traumatic SIRS positive patients was found. Magnesium in high doses may be recommended for traumatic patients with SIRS status to prevent microalbuminuria.

## Background

Magnesium is one of the four most common electrolytes in human body which is an essential co-factor in more than 300 enzymatic reactions. It is involved in many vital processes, such as cardiac excitability [1], transmembrane ion flux, and neurotransmitter release and gating of calcium ion channels [2]. In many respects magnesium serves as a physiological antagonist of calcium [3] and this is a very important theoretical role for magnesium in critical care medicine [4]. Calcium has a defined effect in inflammatory responses, cytokine release and programmed cell death [5]. Magnesium deficiency is common among hospitalized patients (7-11%) and it has been found in 20-60% of intensive care unit (ICU) admitted patients [6]. In near half of electrolyte abnormalities, hypomagnesaemia is found to be a co-morbid factor [7] which indicates its important role in ICU patients [4]. Microalbuminuria is defined as excretion of albumin in small amounts (between 30 and 300 mg/day) that indexed for reliability as microalbumin to creatinine ratio (MACR) [8]. In the past few years, microalbuminuria is utilized for determining severity of inflammatory cascade in ICU patients [9]. It is hypothesized that increasing pro-inflammatory and inflammatory factors will lead to increase in permeability of endothelium and consequent increase of albumin excretion through kidneys.

The relationships between oxidative stress, inflammation, proteinuria and hypomagnesaemia have been evaluated in previous animal and human studies [3,10-12] and cultured human cells [13]. However, we have not came across any study which was conducted in normomagnesemic patients, therefore the effectiveness of magnesium as a therapeutic agent in critical care medicine has not been validated [12,14]. It is hypothesized that high doses of magnesium infusion would be effective and safe for the management of oxidative stress and inflammation following SIRS/trauma.

## Methods

### Study design and setting

This randomized clinical trial study was conducted during 14 months at a University teaching hospital ICU. Block randomization method used to randomize the patient and both patient and researcher were blinded and a third party decides the treatment group designated to patients based on a randomization table.

### Patient population

The study procedure was approved by the University ethics committee with code number (87-03-33-7752) according to the declaration of Helsinki. Patients or their relatives were informed of the study and a written consent was obtained from each individual. Severely ill patients who were admitted for multiple trauma with an Acute Physiology And Chronic Health Evaluation (APACHE II) score of more than 18 accompanied by SIRS criteria [15]. A normal ionized serum magnesium (isMg) level (0.75 < isMg < 0.95 mmol/l) [4] and albumin concentration (40–60 g/l) were the other criteria for acceptance of patients into clinical study pool.

The patient exclusion criteria were conditions that may cause pathological microalbuminuria (creatinine >1.5 mg/dl, metabolic syndrome and diabetes mellitus [16]). Patients with such conditions either before admitting or during stay at ICU were excluded from the study. Patients who had taken drugs which are known as endothelial active drugs (like angiotesin-converting-enzyme, C reactive proteins, corticosteroids and statins) [17], previous magnesium intake, hematologic diseases, neuromuscular disease, pregnancy, severe sepsis and septic shock also were also excluded.

Patients’ ionized serum magnesium concentration was based for determination of magnesium status [18] and all of the patients considered not having serum hyper or hypomagnesaemia at the time of study enrollment. The ionized serum magnesium concentration was measured with ion selective electrodes by Stat Profile Critical Care Xpress analyzer (NOVA biomedical, Waltham, Massachusetts, USA).

Before enrollment a 5 ml blood sample was taken from a central catheter with a heparinized acid washed syringe. This sample was centrifuged in an acid washed test tube and serum derived from blood was used to assess isMg in sample. Before starting infusion and after 6, 18 and 36 hour blood samples were taken in order to measure TNF-α, total antioxidant power and lipid peroxidation.

Total antioxidant power was measured by ferric reducing ability of plasma (FRAP) and the extent of lipid peroxidation was determined with thiobarbituric acid (TBA) method called TBA reactive substances (TBARS) as both were described in our previous paper [1]. TNF-α was assessed with Enzyme-Linked ImmunoSorbaent Assay (ELISA) kit obtained from (BenderMed Systems, Austria) according to manufacturer manual. Creatinine in urine samples were measured with an immunoassay method utilizing BT3500 analyzer (Biotecnica instruments, Italy) by using Jaffe method [19] according to the manufacturer’s brochure. Detection range was >3 mg/L. MACR was determined by ratio between urinary albumin and creatinine concentration. All the samples were tested twice in order to achieve a higher grade of precision. The average of both results were recorded and utilized in analysis.

Patient’s demographic characteristics were recorded at the admission to ICU. The APACHE II scores for definition of severity of illness, and Sequential Organ Failure Assessment (SOFA) score for organ failure were calculated in the first day of admission. Therapeutic intervention scoring system (TISS) score was used to evaluate equal medical treatment among study groups. Deep tendon reflexes were checked hourly during infusion and routinely in the first three days of study. All the patients had urinary catheters and were under mechanical ventilation.

Patients were randomized in this study. High and moderate dose group received 70 or 35 ml of 20% MgSO_4_ solutions, respectively. This amount is equivalent to 15 or 7.5 g of magnesium sulfate for high or moderate group which was diluted into 1000 ml of NaCl (0.9% v/v) and infused in 4 hours.

Three 5 ml midstream urine samples were obtained during the first two days of study. The samples were taken at the time of enrollment (0 hour), 2, 6 and 36 hours post infusion. The study period proceeded for 28 days.

### Statistical analysis

Data are presented as mean with standard errors. Univariate comparisons of baseline characteristics were performed by the independent Student’s t-test. Pearson’s χ2 was used to test differences in survival among groups. Two-way analysis of variance was applied for comparing the repeated measurements of microalbumin levels among groups with multiple comparisons (Scheffe’s test) for each time period. A *p* value of less than 0.05 has been considered statistically significant. Values for microalbumin were log transformed to obtain proportionally constant variation and normally distributed data.

## Results

### Population Characteristics

53 trauma patients enrolled in this study and 8 were excluded. Four of these patients (2 moderate doses, 1 high dose and 1 control) dropped out due to their progression to acute renal injury, 1 patient died during study and 3 patients had developed severe sepsis manifestations. The patients (n = 45) were randomly divided into three groups. The two experimental groups consisted of high and medium level doses of magnesium. The remaining 15 patients were included in a placebo control group. Patient’s demographic characteristics and clinical manifestation are summarized in Table 1. Subjects had no statistical differences in their age, sex, TNF-α, FRAP, TBARS, MACR, APACHE II and SOFA scores at the time of enrollment into the study.

**Table 1:** Demographic data, Scoring systems and MACR at the beginning of the study

		**Control**	**moderate dose**	**High dose**	**Sig.**
Age(years)		49.33333 ± 5.30	39.86667 ± 5.51	48.33333 ± 5.29	NS
sex	Male	13 (86%)	13 (86%)	13 (86%)	NS
	Female	2 (14%)	2 (14%)	2 (14%)	NS
Mortality rate		6 (40%)	7 (46.7%)	5 (33.3%)	NS
APACHE II(0 h)		21.06 ± 0.69	21.20 ± 0.56	21.26 ± 0.58	NS
SOFA(0 h)		8.33 ± 0.30	8.53 ± 0.44	8.20 ± 0.29	NS
TNFα (pg/ml)(0 h)		36.37 ± 11.77	34.16 ± 9.99	32.71 ± 11.42	NS
TBARS (μM)(0 h)		3.89 ± 0.65	4.27 ± 1.44	3.76 ± 1.32	NS
FRAP (μM)		402.44 ± 65.33	399.61 ± 71.83	448.87 ± 85.03	NS
MACR(0 h)*		30.21 ± 5.69	27.59 ± 5.35	29.53 ± 5.35	NS

APACHE = Acute Physiology and Chronic Health Evaluation, SOFA = Sequential Organ Failure Assessment, MACR = microalbumin to creatinine ratio. * mg/mmol.

It is important to note that patients ionized serum magnesium concentration was based for determination of magnesium status [18]. This method is considered to be the most accurate (Table 1). All the patients’ baseline serum ionized magnesium concentration was in normal range at the start point of the study.

### Effect of magnesium on oxidative & inflammatory factors

The TNF alpha levels remained steady for the control group at all time points. There was only a slight (statistically insignificant) decrease in TNF-α at 36-hour time point for both experimental groups (Table 2), suggesting that the blood retained its reductive characteristics. The level of FRAP remained steady for all the groups. However, TBARS level for the high dose group demonstrated a considerable drop in readings at 36 hour (3.0 ± 1.0) when it was compared to the 18 hour readings (3.6 ± 1.2) for the high dose group. The reduction in TBARS level is an indication of the positive effect of high dose of magnesium on traumatic patients who were positive for systemic inflammatory response syndromes (SIRS) normomagnesemic status (Table 2).

**Table 2:** Inflammatory and oxidative factors

		**Control group**	**Medium dose group**	**High dose group**
TNF-α pg/ml ± SD	0 h	36.0 ± 12.1	37.0 ± 12.2	38.1 ± 10.7
	6 h	37.4 ± 13.2	34.5 ± 8.1	35.6 ± 15.4
	18 h	35.6 ± 11.6	36.38 ± 10.8	30.1 ± 7.0
	36 h	36.3 ± 10.9	28.6 ± 6.5	26.9 ± 7.9
FRAP μM ± SD	0 h	421. ± 54.5	412.3 ± 69.9	424.7 ± 71.4
	6 h	397.1 ± 69.0	398.7 ± 73.0	427.5 ± 74.6
	18 h	407.7 ± 82.2	401.8 ± 90.7	452.7 ± 90.8
	36 h	383.4 ± 51.4	385.5 ± 53.8	490.5 ± 92.5
TBARS μM ± SD	0 h	4.1 ± 0.7	4.3 ± 1.7	4.4 ± 1.0
	6 h	3.7 ± 0.5	4.4 ± 1.0	3.9 ± 1.5
	18 h	3.9 ± 0.7	4.2 ± 1.1	3.6 ± 1.2
	36 h	3.7 ± 0.4	4.1 ± 1.4	3.0 ± 1.0

**Figure 1:**
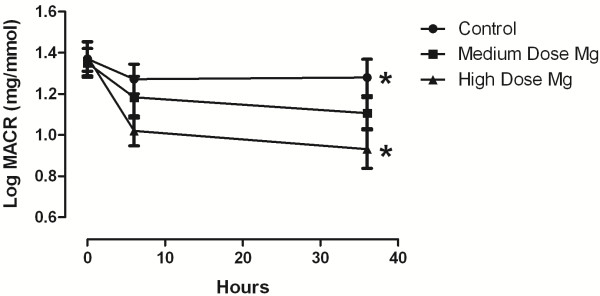
**Changes in microalbumin/creatine in urine samples of control group vs. moderate dose and high dose magnesium group by hours.** * p < 0.05 vs high dose magnesium group.

There were no significant difference in levels of TNF- α and TBARS. Another noticeable change was microalbuminuria, at 36 hours post magnesium infusion in high dose group which was determined to be statistically lower than the control group (p = 0.024)(Figure 1). The trends of other mentioned anti inflammatory and oxidative factors proved our hypothesis on existence of lower levels of MACR over time between high dose infusion and placebo groups.

### Outcome

A trend toward mortality reduction was found in high dose magnesium recipients although this difference was not statistically significant. Patients tolerated MgSO_4_ infusion very well with no significant difference between treatment and placebo groups (p = 0.61) and no serious adverse reaction were reported, except for 2 cases of hypermagnesaemia in the high dose group which did not have any clinical consequences.

## Discussion

This study demonstrate that high doses of magnesium reduce microalbuminuria in traumatic critically ill patients at 36 hour post infusion. Although no significant difference observed among trends, there was a positive trend toward better outcomes in treatment groups in total antioxidant power, lipid peroxidation and TNF-α between treatment and placebo groups.

Microvascular changes induced by nitric oxide (NO) and other proinflammatory factors and their interaction with leukocytes amplify inflammatory response in SIRS patients. Endothelial cells could be injured through inflammatory response and cause an increase in capillary permeability particularly in glomerular vessels [8] which induces kidneys to undergo transient proteinuria. The deleterious scale of these changes can be measured by increased levels of microalbuminuria [8]. The population enrolled in this study was homogenous and consisted of neurotraumatic critically ill only. The purpose of this study is to evaluate the efficacy and rational of magnesium infusion on neurotraumatic patients. In traumatic condition, an energy depletion dilemma emerges in cellular level. The consequence of this depletion is reduction in activity of transmembrane calcium ion transport channels which leads to distorted ion balance and permeability changes. Membrane permeability changes cause calcium influx in cells, which results in many deleterious reactions such as alternation of adenosine triphosphate (ATP) production in mitochondria, overproduction of reactive oxygen species (ROS), NO generation and release of proinflammatory factors [1,2]. Magnesium could serve as antagonist of calcium to inhibit these harmful effects [3] in traumatic patients.

In previous animal studies it was demonstrated that hypomagnesaemia can cause systemic inflammatory syndrome [12]. Magnesium deficiency opens N-methyl-D-aspartate (NMDA) calcium channels and activates nuclear factor-kappa B (NF-kB) as primary mechanism of inflammation [11]. However, in this study after magnesium infusion, the levels of TNF-α, total antioxidant power and lipid peroxidation did not demonstrate any significant change at various periods of analysis. Therefore, administration of magnesium salt did not display any anti-inflammatory activities in critically ill patients with normal levels of this ion. Effect of body magnesium levels (plasma, erythrocyte and urine) and its association with microalbuminuria has been previously studied on type 1 diabetic patients [20]. A negative relation between erythrocyte magnesium level and microalbuminuria was observed suggesting hypomagnesaemia as a risk factor for proteinuria in diabetic patients. In another study, it was suggested that hypomagnesaemia is an independent risk factor for microalbuminuria in type two diabetic patients [20]. In the present study there has been a lag about 48–72 hour between hospital and ICU admission that might be the time that most critical changes in level of TNF-α and oxidative marker occurred and thus less significant changes were observed subsequently. As we mentioned in previous study with cytokines determination of cutoff point for cytokine measurement in ethnic populations remained unclear and problematic [21]. Our previous study verified that N-acetylcysteine performs as a free radical scavenger and an anti-inflammatory agent and improves microalbuminuria in acute respiratory distress syndrome patients [22]. It has been illustrated that severity of microalbuminuria has a direct relationship with mortality and morbidity rates [9,16]. Although, no significant change in TNF-α and oxidative factors showed up in the study time window, the change in microalbumin suggests that inflammation has been reduced in the high dose treatment subjects. In order to omit the bias of hypomagnesaemia effect on proteinuria [20], patients with normal levels of serum magnesium were allowed to be enrolled in this clinical study. Magnesium total serum concentration is the most studied laboratory parameter and considered poorly related to cellular status of magnesium in the patient’s body. Magnesium loading test or ionized serum magnesium concentration is used for evaluation of magnesium sufficiency in the body [23]. Magnesium loading test was not utilized for evaluation of magnesium status in this study because patients received a large dose of magnesium before enrollment in the study and the result would be compromised and unreliable. Therefore, ionized serum magnesium test was utilized in order to establish magnesium status. Ionized serum magnesium test has been applied to establish magnesium status instead of magnesium loading test due to limitation of previous magnesium intake. In this study, the serum albumin concentration was assessed prior to the evaluation of magnesium status. High levels of albumin may interfere with ionized serum magnesium test [23] and may negatively impact the accurate evaluation for microalbuminuria.

Although, management of patients in ICU is somehow complicated and dependent of the status of each patient [24], it was decided to use an established protocol as a base amount for moderate dose of magnesium that was safe over the years in ICU [25]. Two fold of that dose has been assumed as high dose of magnesium for this study. The positive effect of magnesium dosing in critically ill patients is under debate and the results are controversial. Determination of a specific dose was difficult because compensation for magnesium depletion was not the aim of this study. The normomagnesemic populations have never been studied before to the extent of our knowledge. Therefore, clinically significant results would be due to the theoretical role of magnesium. There were no significant difference among groups during time, but after 36 hours, the MACR in high dose magnesium group was significantly lower than control group (Figure 1).

Mild bradycardia and hypotension were the only side effects observed in patients who received magnesium. Therefore we suggest that magnesium doses as high as the ones used in this study are relatively safe for infusion of critically ill patients. However magnesium therapy should be considered in special populations. Magnesium is generally cleared by kidneys and magnesium therapy in higher doses should be administered with more caution in patients with any risk of renal failure.

Administration of magnesium as a therapeutic agent is not a common approach, but it is routinely employed to correct the hypomagnesemic state. In this study magnesium sulfate infusion was administered as an anti-inflammatory therapeutic agent and some optimistic results were obtained. In order to obtain a more definitive conclusion, another more elaborated clinical trial is recommended.

There was a beneficial trend toward high magnesium load where we left the study (Figure 1). The number of subjects and the duration of study were limited in this trial. The results could not be generalized to the population in this condition, but it is recommended to conduct more studies with longer duration and greater sample size. It is also recommended to infuse magnesium in longer duration and more divided intervals.

## Conclusion

We did not find conclusive evidence for antioxidative and antiinflammatory effects of magnesium in traumatic SIRS positive patients. However, high dose magnesium might be recommended as a safe option for preventing microalbuminuria in traumatic patients with SIRS criteria.

## Abbreviations

ICU: Intensive care unit; SIRS: Systemic inflammatory response syndromes; TNF-α: Tumor necrosis factor alpha; MACR: Microalbumin to creatinine ratio; APACHE: II: Acute Physiology And Chronic Health Evaluation; isMg: Ionized serum magnesium; FRAP: Ferric reducing ability of plasma; TPTZ: 2,4,6 Tripyridyl-s-Triazine; HCl: Hydrochloric acid; TBARS: Thiobarbituric acid reactive substances; TCA: Trichloroacetic acid; ELISA: Enzyme-Linked ImmunoSorbaent Assay; SOFA: Sequential Organ Failure Assessment score; TISS: Therapeutic intervention scoring system; ROS: Reactive oxygen species; NO: Nitric oxide; NMDA: N-methyl-D-aspartate; NF-kB: Nuclear factor-kappa B; MLT: Magnesium loading test.

## Competing interests

There are no conflicts of interest related to this publication.

## Authors’ contributions

BM and HH were responsible for drafting of the article, and gathering and analysis of data. AA, AN and MRM were responsible for patient inclusion, study design, implementation of study and gathering the data. MAM was responsible for analyzing the samples and laboratory data. MA shared in the idea and edited the paper. MM was responsible for conception of study, critical revision and drafting the article. All authors read and approved the final manuscript.
